# The Role of Munc18-1 and Its Orthologs in Modulation of Cortical F-Actin in Chromaffin Cells

**DOI:** 10.1007/s12031-012-9775-8

**Published:** 2012-04-26

**Authors:** Julia Kurps, Heidi de Wit

**Affiliations:** Department of Functional Genomics and Clinical Genetics, Center for Neurogenomics and Cognitive Research, Neuroscience Campus Amsterdam, VU University Amsterdam and VU University Medical Center, De Boelelaan 1085, 1081 HV Amsterdam, The Netherlands

**Keywords:** Docking, Exocytosis, Chromaffin cell, Munc18, Syntaxin-1, F-actin

## Abstract

Munc18-1 was originally described as an essential docking factor in chromaffin cells. Recent findings showed that Munc18-1 has an additional role in the regulation of the cortical F-actin network, which is thought to function as a physical barrier preventing secretory vesicles from access to their release sites under resting conditions. In our review, we discuss whether this function is evolutionarily conserved in all Sec1/Munc18-like (SM) proteins. In addition, we introduce a new quantification method that improves the analysis of cortical filamentous actin (F-actin) in comparison with existing methods. Since the docking process is highly evolutionarily conserved in the SM protein superfamily, we use our novel quantification method to investigate whether the F-actin-regulating function is similarly conserved among SM proteins. Our preliminary data suggest that the regulation of cortical F-actin is a shared function of SM proteins, and we propose a way to gain more insight in the molecular mechanism underlying the Munc18-1-mediated cortical F-actin regulation.

## Role of Munc18-1 in Docking in Chromaffin Cells

Regulated exocytosis of large dense-core vesicles (LDCV) is an essential process in chromaffin cells. Here, hormones and neuropeptides, which are stored in LDCVs, are released into the bloodstream. The initial step of vesicle secretion is the morphological docking of LDCVs to the plasma membrane, which is followed by priming and finally fusion of the vesicles. One protein family which is known to be essential for docking and fusion is the highly evolutionary conserved Sec1/Munc18-like (SM) protein family. The absence of Sec1, the Munc18-1 ortholog in yeast (*Saccharomyces cerevisiae*), leads to a secretion defect and to accumulation of secretory vesicles near fusion sites (Novick et al. 1981; Tschopp et al. 1984). Similarly, Rop (Sec1 ortholog in *Drosophila melanogaster*) and Unc18 (Sec1 ortholog in *Caenorhabditis elegans*) null mutants show a reduction of docked secretory vesicles at their release sites (Schulze et al. 1994; Weimer et al. 2003). The first proteins that were shown to be essential for the docking of secretory vesicles in mammals were Munc18-1 (Voets et al. 2001) and syntaxin-1 (De Wit et al. 2006). Both proteins and their essential role in the docking process are highly conserved from yeast to mammals.

The nature of the interaction of syntaxin-1 and Munc18-1 is complex, since Munc18-1 can bind syntaxin-1 in two distinct modes. Munc18-1 can either bind the “open” syntaxin-1 in the assembled soluble *N*-ethylmaleimide-sensitive factor attachment receptor (SNARE) complex or the “closed” conformation of isolated syntaxin-1 (Pevsner et al. 1994; Dulubova et al. 2007; Khvotchev et al. 2007; Burkhardt et al. 2008; Gerber et al. 2008). The two distinct binding modes reflect distinct functions of Munc18-1. Two recent findings support the hypothesis that the binding of Munc18-1 to the closed conformation is responsible for the docking mechanism: (1) expression of an open syntaxin-1 mutant which only allows N-terminal interaction with Munc18-1 in an assembled SNARE complex showed a severe docking phenotype (Gerber et al. 2008) and (2) the robust docking phenotype in Munc18-1 *null* chromaffin cells was partially rescued when a Munc18-1 mutation, which is known to perturb the binding with the “closed” syntaxin-1 conformation, was expressed (De Wit et al. 2009). Those findings led to the conclusion that the binding of Munc18-1 to the “closed” conformation of syntaxin-1 is the functional interaction involved in the docking process, whereas the binding of Munc18-1 to the “open” syntaxin-1 in the assembled SNARE complex seems to be essential for later exocytosis steps such as fusion (Dulubova et al. 2007; Khvotchev et al. 2007; Barclay 2008; Burkhardt et al. 2008).

According to the current docking model obtained from electron microscopy studies on adrenal chromaffin cells from genetically modified mouse embryos, four proteins are involved in the formation of the minimal docking machinery. Those four proteins are syntaxin-1, Munc18-1, synaptosomal-associated protein 25 (SNAP-25), and synaptotagmin-1. The initial step of the vesicle docking is the formation of a 1:1 acceptor complex at the target membrane, containing the target soluble *N*-ethylmaleimide-sensitive factor attachment receptors’ (t-SNAREs) syntaxin-1 and SNAP-25 (De Wit et al. 2009). Munc18-1 stabilizes this acceptor complex probably via binding to syntaxin-1. Recent findings showed that the vesicular protein synaptotagmin-1 binds to the acceptor complex and thereby anchors secretory vesicles to their docking sites at the plasma membrane (Söllner et al. 1993; Schiavo et al. 1997; Chieregatti et al. 2002; Rickman et al. 2004; De Wit et al. 2009). It is now widely accepted that the interaction of Munc18-1 with the “closed” syntaxin-1 conformation is essential for the docking process, whereas its binding to the “open” conformation seems to be involved in a suggested postdocking role of Munc18-1. This function of Munc18-1 will not be discussed here. For detailed reviews and the molecular mechanism underlying the role of Munc18-1 in the docking process in adrenal chromaffin cells, see Verhage and Sørensen (2008) and De Wit (2010a, b).

## Role of Cortical F-Actin in Regulated Secretion in Chromaffin Cells

In addition to the minimal docking machinery (syntaxin-1, SNAP-25, synaptotagmin-1, and Munc18-1), the dense cortical network of filamentous actin (F-actin) underneath the plasma membrane was shown to mediate regulatory secretion in chromaffin cells (Aunis and Bader 1988; Trifaró et al. 1992). Since stimulation of chromaffin cells by high K^+^, phorbol esters, or nicotine resulted in a decrease in the integrity of the cortical F-actin network and an enhanced release of hormones and neuropeptides, it is suggested that this network forms a physical barrier for secretory vesicles (Trifaró et al. 1992). Under resting conditions, the F-actin network is highly polymerized and secretory vesicles cannot reach the plasma membrane, whereas stimulation leads to a depolymerization of the cortical F-actin, so secretory vesicles gain access to their release sites (Vitale et al. 1995). Since the known regulatory mechanisms, including essential proteins that control cortical F-actin dynamics, were the topic of several earlier reviews (Doussau and Augustine 2000; Trifaró et al. 2000; Trifaró et al. 2008), they will not be discussed here. However, the analysis methods which were used to access alterations in the cortical F-actin network will be shortly discussed. In the first studies, populations of neuroendocrine cells were classified according to the appearance of the rhodamine–phalloidin staining of the cortical F-actin. Here, it was distinguished whether the cortical staining of single cells was continuous or composed of discontinuous patches and the changes in the F-actin network were presented as the percentage of cells with a discontinuous staining (Trifaró et al. 1992). Another method used alterations in the total intensity of the cortical fluorescence of the rhodamine–phalloidin staining as a measure for changes in the cortical F-actin (Doreian et al. 2008). Both methods focus on the overall F-actin content of chromaffin cells and on the changes during stimulations or manipulations. However, for our observations, we were interested in the subcellular localization of F-actin patches along the plasma membrane and, as such, in a more precise quantification of the cortical F-actin network.

## Quantification of Cortical F-Actin in Chromaffin Cells

**Fig. 1:**
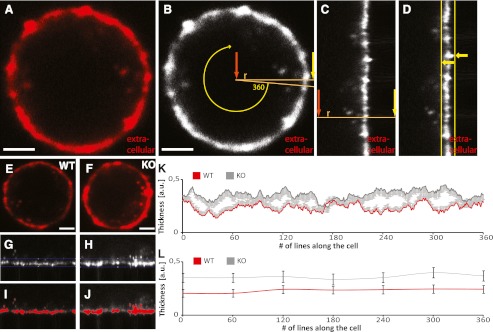
Cortical F-actin quantification and alterations in thickness of cortical F-actin network due to absence of Munc18-1. **a** Cortical F-actin in chromaffin cell (fixed with 4 % PFA at DIV3), stained with rhodamine–phalloidin and imaged with confocal microscopy. **b** Visualization of polar transformation of the rhodamine–phalloidin signal. **c** Rectangular signal after polar transformation. **d** Automatic, threshold-based definition of region of interest. **e**, **f** Cortical F-actin in Munc18-1 wild type and Munc18-1 null chromaffin cell. **g**, **h** Automatically defined region of interest of **e** and **f**. **i**, **j** Pixels with intensity values above threshold of **g** and **h**. **k** Quantification of cortical F-actin thickness and comparison between Munc18-1 wild type (*n* = 25) and Munc18-1 null (*n* = 25) chromaffin cells. **l** Binned (bin size = 60 values) thickness values of **k**. *Scale bars*, 2 μm

In order to describe our observation of Munc18-1-dependent F-actin regulation in detail, a reliable F-actin quantification method was required. Therefore, we developed an algorithm, which is performed in the image analysis program ImageJ and which enables us to quantify the alterations in cortical F-actin under various conditions. We use confocal microscopy images of fixed chromaffin cells which are stained with rhodamine–phalloidin to visualize the cortical F-actin network underneath the plasma membrane (Fig. 1a, e, f). By using a process called polar transformation, the circular signal (Fig. 1a, b) is translated into a rectangular signal (Fig. 1c), which simplifies the threshold-based detection of the region of interest. For the polar transformation, the program first determines the center of the cell and generates a line from this point to the edge of the image. The number of pixels along the line depends on the size of the image. This step is repeated 360 times, with every new line being generated in an angle of 1 ° from the previous line (Fig. 1b). The 360 lines, covering all pixels in the original image, form a new, transformed image (Fig. 1c). Subsequently, the imageJ analysis program automatically determines the right (extracellular) border of the cortical F-actin network, based on a threshold, which is dependent on the intrinsic image properties. The left (intracellular) border of the F-actin network is fixed as 40 pixels left from the right border (Fig. 1d). This measure was determined manually, since the automatic detection of the intracellular border is difficult, due to intracellular staining. The choice for the width of 40 pixels for the region of interest (ROI) is based on observations of a great amount of chromaffin cells, showing that the rhodamine–phalloidin-based signal does not extend this value. After the ROI is automatically determined, two output sheets are generated. One includes intensity values of all pixels in the ROI, and the other one contains the average signal intensity of all 360 lines as well as their thickness and density. For the described calculations, only pixels with intensity values above the automatically determined threshold are used.

Our new quantification method enables us to characterize the alterations in the cortical F-actin network due to the absence or presence of Munc18-1 in much more detail than in our previous study (Toonen et al. 2006). In Fig. 1e, f, typical examples of the F-actin network in Munc18-1 *wild type* and Munc18-1 *null* chromaffin cells are shown, clearly indicating that the absence of Munc18-1 leads to a thicker cortical F-actin network. This observation is clearly detectable in the transformed images (Fig. 1g, h) and reflected by the visualization of all pixels with intensity values above threshold, which are marked in red in Fig. 1i, j. The quantification of the cortical F-actin in populations of Munc18-1 *wild type* (*n* = 25) and Munc18-1 *null* (*n* = 25) chromaffin cells is shown in Fig. 1k. Here, the thickness of the cortical F-actin network is displayed for every line of the transformed image. The thickness is calculated as the sum of all pixels with an intensity value above the threshold divided by 40, which determines the maximal number of pixels in one line of the region of interest. In order to show the increased thickness of the F-actin in Munc18-1 *null* cells even clearer, the thickness values were averaged in bins of 60 values (Fig. 1l). Since cell culture, fixation, immunocytochemical processes, and the image acquisition are highly standardized and identical for both genotypes, the observed effect is solely dependent on the presence of Munc18-1.

## Cortical F-Actin Regulation: Conserved Function of SM Proteins?

In order to clarify the role of Munc18-1 in the regulation of cortical F-actin in chromaffin cells, the evolutionary conservation of this process was questioned. Since actin is also highly conserved during evolution, there is an indication that actin-regulating functions and underlying mechanisms are also conserved during evolutionary development (Bhagavathi and Malathi 1996). Furthermore, the function of Munc18-1 in the docking process is highly evolutionary conserved (Schulze et al. 1994; Weimer et al. 2003, Toonen et al. 2006). Therefore, the question arose whether the F-actin-regulating function of Munc18-1 is similarly conserved and whether other members of the SM protein family can regulate the cortical F-actin in chromaffin cells in the same way as Munc18-1.

**Fig. 2:**
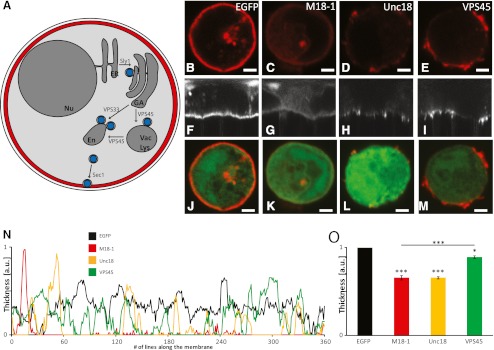
Role of SM proteins in the vesicle cycle and in the regulation of cortical F-actin in chromaffin cells. **a** *Cartoon model* of SM proteins in intracellular vesicle trafficking pathways. The Sly1p subfamily is involved in the transport of secretory vesicles between the endoplasmatic reticulum (*ER*), which is attached to the nucleus (*Nu*), and the Golgi apparatus (*GA*), whereas members of the VPS33p subfamily participate in the vesicle transport to vacuoles (*Vac*) or lysosomes (*Lys*). The VPS45p subfamily regulates endosomal (*En*) vesicle trafficking, and members of the Sec1p family (e.g., Rop in *D. melanogaster*, Unc18 in *C. elegans*, and Munc18 in mammals) are essential for regulated secretion. Visualization of cortical F-actin in chromaffin cells. The images show typical examples of Munc18-1 null chromaffin cells after expression of either EGFP alone (**b**) as control or the SM proteins Munc18-1 (**c**), Unc18 (**d**), and VPS45 (**e**). Acute expression of SM genes was induced with Semliki Forest virus (SFV) from a bicistronic message containing a poliovirus internal ribosomal entry site and EGFP. **f–i** Confocal images of **b**–**e** after polar transformation. **j–m** Confocal images showing the successful SFV infection of the chromaffin cells, since all SM proteins were coexpressed with EGFP to identify infected chromaffin cells. **n** Visualization of typical examples of cortical F-actin thickness along the plasma membrane in Munc18-1 null chromaffin cells which express EGFP or SM proteins. **o** Quantification of average thickness of the cortical F-actin in Munc18-1 null chromaffin cells which express EGFP or SM proteins. For all conditions, *n* = 5 and the *error bars* display the SEM. *p < 0.05, ***p < 0.001 by Student’s *t* test compared to EGFP controls. *Scale bars*, 2 μm

The family of SM proteins consists of four subfamilies, which were defined according to their function in intracellular vesicle-trafficking processes (Fig. 2a).

### Sec1p Family

The Sec1 family is highly evolutionarily conserved, and its members are known to be involved in exocytosis events. The Sec1 family includes the yeast (*S. cerevisiae*) protein Sec1p as well as its homologs in *D. melanogaster*, Rop, and in *C. elegans*, Unc18. The mammalian Munc18 isoforms Munc18-1, Munc18-2, and Munc18-3 are also members of the Sec1 family. Munc18-1 is known to be essential for docking of secretory vesicles to their target membrane but is also believed to have a function in the priming step (Gulyás-Kovács et al. 2007). Munc18-1 was shown to bind the syntaxin isoforms syntaxin-1, syntaxin-2, and syntaxin-3 (Toonen and Verhage 2003), and it is known that the SM protein binds syntaxin in the “closed” formation in the docking mechanism (De Wit 2010a, b). However, Munc18-1 can bind syntaxin in the “open” formation as well (Dulubova et al. 1999). This binding mode is believed to be involved in events downstream of docking (De Wit 2010a, b). The Munc18 isoforms Munc18-2 and Munc18-3 show lower expression in neuroendocrine cells (Hata and Südhof 1995; Tellam et al. 1995). Like Munc18-1, Munc18-2 shows a high affinity for syntaxin-1, syntaxin-2, syntaxin-3 and Munc18-3 shows a high affinity for syntaxin-2 and syntaxin-4 (Hata and Südhof 1995; Tellam et al. 1997).

### VPS45p Family

The VPS45 family contains vacuolar proteins and is important for the regulation of endosomal trafficking (Tellam et al. 1997). In yeast, VPS45p binds to the syntaxin Tlg2p, which plays an essential role in the vesicle transport between the *trans*-Golgi network (TGN) and early endosomes (Dulubova et al. 2002). VPS45p binds the conserved N-terminal sequence of Tlg2. It was recently discovered that VPS45p has an additional binding site, where Tlg2p in its “closed” formation can be bound. This might result in a competition between the N-terminal Tlg2p peptide and “closed” Tlg2p, and therefore, a regulatory mechanism for the SM protein–syntaxin interaction was suggested by the authors (Furgason et al. 2009). VPS45 homologs in *D. melanogaster* and mammals were also described. The mammalian homolog VPS45 binds to syntaxin-16, which is a homolog of Tlg2p and forms a SNARE complex with syntaxin-6 and Vti 1a (Dulubova et al. 2002). The VPS45–syntaxin-16 interaction was shown to be involved in TGN transport, leading to the conclusion that the mechanism of vesicle trafficking between the TGN and early endosomes is highly conserved (Bassham et al. 2000; Struthers et al. 2009). VPS45p was also found to participate in late-endosomal vesicle transport, which is dependent on the syntaxin Pep12p. Interestingly, until now, no direct binding between Pep12p and VPS45p was observed (Webb et al. 1997).

### Sly1p Family

Members of the Sly1p family are hydrophobic proteins, which regulate vesicle trafficking between the endoplasmic reticulum (ER) and the Golgi network as well as the retrograde transport back to the ER (Li et al. 2005). Furthermore, a Sly1p function in intra-Golgi transport mechanisms was described. Sly1 binds to an evolutionary conserved N-terminal peptide motif of the Golgi t-SNARE Sed5 and the ER t-SNARE Ufe1 in yeast (Søgaard et al. 1994). More recent findings showed that the binding of Sly1 to Sed5 enhances the formation of the trans-SNARE complex (Kosodo et al. 2002). Noteworthy, the mammalian Sly1 ortholog binds to the same N-terminal peptide motif of syntaxin-5 and syntaxin-18 in vertebrates (Yamaguchi et al. 2002).

### VPS33p Family

The VPS33p subfamily of SM proteins was first shown to regulate the vesicle transport to vacuoles (Banta et al. 1990) and has an additional function in vesicle-trafficking processes between the late Golgi and the endosome (Subramanian et al. 2004). It was shown that VPS33p interacts with the endosomal syntaxin Pep12, which was previously identified as a binding protein of the SM protein VPS45 (Cowles et al. 1997). This is the only demonstrated interaction between an individual syntaxin and two SM proteins at the same organelle. Furthermore, VPS33 was found to be involved in the transport of endocytosed vesicles to lysosomes (Akbar et al. 2009).

## Preliminary Data

In order to analyze whether SM proteins, other than Munc18-1, affect the cortical F-actin network in chromaffin cells, we performed pilot experiments. We infected adrenal chromaffin cells from embryonic Munc18-1 *null* mice with Semliki Forest virus constructs of the SM proteins Unc18 and VPS45. The cultured chromaffin cells were fixed with 4 % paraformaldehyde (PFA) at DIV3 (6 h after the viral infection), and the cortical F-actin was stained with rhodamine–phalloidin. Images were acquired with confocal microscopy and analyzed with our newly developed quantification method. We determined whether the overexpression of the two described SM proteins, participating in different intracellular vesicle-trafficking processes (Unc18, vesicle transport to plasma membrane; VPS45, vesicle transport to lysosomes and endosomes), leads to alterations in the cortical F-actin which are similar to the changes we observed after the overexpression of Munc18-1.

Our preliminary results show that both SM proteins can regulate the thickness of cortical F-actin in chromaffin cells, independently from the pathways in which they are known to participate in. As shown in Fig. 2b, the rhodamine–phalloidin staining of Munc18-1 *null* chromaffin cells expressing enhanced green fluorescent protein (EGFP) shows a similar thick cortical F-actin network as uninfected controls described previously (Toonen et al. 2006 and Fig. 1). The overexpression of the SM proteins Munc18-1, Unc18, and VPS45 via Semliki Forest virus infection resulted in a decrease in cortical F-actin, as shown in Fig. 2c–e. This effect is reflected in the transformed images (Fig. 2f–i) as well as in the graphs in Fig. 2m, n. Typical examples of the cortical F-actin distribution along the plasma membrane in all four conditions are visualized in Fig. 2m. The average thickness of the network, dependent on the presence of the described SM proteins, is shown in Fig. 2n. It becomes clear that the overexpression of *wild type* Munc18-1 led to the strongest effect (35 % reduction in F-actin thickness) and a complete rescue of the Munc18-1 *null* phenotype. The decrease in cortical F-actin thickness, observed after the overexpression of Unc18 (35 % reduction in F-actin thickness), is similar to the effect of overexpression of *wild type* Munc18-1. This result can be expected, since Unc18 is closely related to its mammalian ortholog Munc18-1 (amino acid sequence homology to Munc18-1 according to Clustal alignment, 57 %). Furthermore, Unc18 is known to be involved in the secretory vesicle trafficking to the plasma membrane and essential for the docking process. Surprisingly, the overexpression of the SM protein VPS45 (amino acid sequence homology to Munc18-1 according to Clustal alignment, 18 %), which is important for vesicle transport to lysosomes and endosomes, seems to have a regulating effect on the cortical F-actin (11 % reduction in F-actin thickness) as well. However, compared to the overexpression of Munc18-1 *wild type* or Unc18, the rescue of the phenotype observed in Munc18-1 *null* chromaffin cells was less prominent after the overexpression of VPS45.

## Discussion and Perspectives

In our review, we describe the well-known role of Munc18-1 in docking of secretory vesicles to the plasma membrane and its unresolved function in the regulation of cortical F-actin in chromaffin cells. In addition, we shortly discuss the previously used F-actin quantification methods in comparison to our novel analysis algorithm. In earlier studies, overall changes in the cortical F-actin network, due to stimulation or genetic manipulations, were determined and presented as the percentage of cells with altered cortical F-actin. However, with our newly developed quantification method, we are able to analyze the localization and distribution of F-actin along the plasma membrane in a more detailed fashion. This method, together with immunohistochemistry approaches, allows us to also investigate colocalization of F-actin patches and actin-regulating proteins, which will help to unravel the function of Munc18-1 in the regulation of this subplasmalemmal network. One way to investigate the molecular mechanism that underlies this process is to analyze whether Munc18-1 orthologs, which belong to the SM protein family, show the same ability as Munc18-1 to rescue the Munc18-1 *null* phenotype by decreasing the cortical F-actin network to the level of Munc18-1 *wild type*. The four subfamilies of this protein family are shortly described in this review. In order to test their F-actin-regulating function, we overexpressed two SM proteins in chromaffin cells with a Munc18-1 *null* background, one which participates in the vesicle trafficking to the plasma membrane (Unc18) and one which is involved in vesicle transport to lysosomes and endosomes (VPS45). Both SM proteins are able to downregulate the thick cortical F-actin network that we observe in Munc18-1 *null* chromaffin cells. However, the rescue effect on the F-actin thickness after the expression of Unc18 or VPS45 is not as dramatic as after the expression of *wild type* Munc18-1. The only common domain of all SM proteins is the Sec1 like domain. However, this is not enough to explain our observations, since the amino acid sequences of the SM proteins in this domain show a great variability. We believe that an elaborate sequence and domain analysis might be one way to understand the function of SM proteins in the regulation of F-actin. There is no evidence for a direct interaction of this protein family and the cytoskeletal component, but there are hints that point to distinct pathways. One interesting observation by Morgera et al. (2011) showed that the yeast SM protein Sec1 can directly bind to proteins of the exocyst complex and that exocyst subunits interact with the yeast ortholog of myosin V, Myo2 (Jin et al. 2011). This finding, like our observations, links essential parts of the secretory machinery to components of the cytoskeleton. Another observation connects the SM protein VPS45 to Rab27, which was shown to operate in a complex with myosin Va, which in turn interacts directly with actin filaments (Fukuda 2003). Further experimental efforts will be necessary to unravel the exciting new connection between SM proteins and subplasmalemmal actin cytoskeleton.

## Abbreviations

ER: Endoplasmatic reticulum
F-actin: Filamentous actin
LDCV: Large dense-core vesicle
PFA: Paraformaldehyde
PKC: Protein kinase C
ROI: Region of interest
SM: Sec1/Munc18-like
SNARE: Soluble *N*-ethylmaleimide-sensitive factor attachment receptor
TGN: Trans-Golgi-network
